# Toxicity Mechanism of Chlorinated Paraffins with Different Carbon Chain Lengths to *Chlorella* sp. and *Microcystis aeruginosa*

**DOI:** 10.3390/toxics14040311

**Published:** 2026-04-04

**Authors:** Qihui Li, Jue Li, Guo Li, Peng Lin, Sen Liu, Lin Deng, Yangjinzhi Yu, Xiaowei Zheng, Weizhen Zhang, Zhengqiu Fan

**Affiliations:** 1 Department of Environmental Science & Engineering, Fudan University, Shanghai 200438, China; 2 Department of Evolutionary Stress Ecology & Ecotoxicology, Leuven University, 3000 Leuven, Belgium; 3 College of Ecology and Environment, Chengdu University of Technology, Chengdu 610059, China; 4 School of Global Health, Chinese Center for Tropical Diseases Research, Shanghai Jiao Tong University School of Medicine, Shanghai 200025, China; 5 Sichuan Fuhai Origin Ecological Technology Co., Ltd., Chengdu 610213, China; 6 Sichuan Jinshadao Biotechnology Co., Ltd., Chengdu 610059, China

**Keywords:** chlorinated paraffins, eukaryotic algae, prokaryotic algae, toxicity mechanisms

## Abstract

Chlorinated paraffins (CPs) are widely used, structurally complex mixtures of chlorinated alkanes whose ecological risks in aquatic ecosystems have raised increasing concern. However, the toxic effects and molecular mechanisms of CPs on primary aquatic producers remain poorly understood. In this study, we used the eukaryotic green algae *Chlorella* sp. and the prokaryotic cyanobacterium *Microcystis aeruginosa* (*M. aeruginosa*) as test organisms to systematically investigate the effects of CPs with different carbon chain lengths, namely short-chain CPs (SCCPs), medium-chain CPs (MCCPs), and long-chain CPs (LCCPs), on algal growth, photosynthetic pigment content, antioxidant systems, cellular ultrastructure, and the underlying molecular responses. Our results showed that CPs toxicity to algae is significantly dependent on both CPs carbon-chain length and algal species. Exposure to 1.0 mg/L SCCPs for 96 h produced a growth inhibition of *Chlorella* sp. of 14.45%. CPs’ exposure significantly altered algal Chl-a content and elicited antioxidant defense responses, and affected the synthesis and extracellular release of MC-RR and MC-LR in *M. aeruginosa*. Ultrastructural observations revealed cell surface wrinkling and deformation in both *Chlorella* sp. and *M. aeruginosa*. *Chlorella* sp. additionally exhibited thylakoid disintegration and plasmolysis. Transcriptomic analysis indicated that CPs with different chain lengths significantly downregulated genes in *Chlorella* sp. associated with DNA replication and mismatch repair, suggesting impairment of replication initiation and elongation and compromised genome stability. Concurrently, genes encoding photosynthetic antenna proteins and carbon fixation were upregulated. In *M. aeruginosa*, CPs exposure markedly disturbed energy metabolism pathways, including glycolysis/gluconeogenesis and oxidative phosphorylation, which were generally downregulated. This study provides a comparative assessment of CPs’ toxicity between the eukaryotic algae *Chlorella* sp. and the prokaryotic algae *M. aeruginosa*, revealing that toxicity is co-determined by carbon chain length and algal species. Additionally, it provides critical toxicological data and establishes a theoretical foundation for the scientific assessment of the aquatic ecological risks posed by CPs with different carbon chain lengths.

## 1. Introduction

Chlorinated paraffins (CPs), with the general molecular formula C_x_H_(2x−y+2)_Cl_y_ (where x = 10–30, y = 1–17), possess chlorine contents ranging between 30.0% and 70.0%. As a class of complex synthetic organochlorine compounds, CPs are widely used in plastics, rubber, coatings, and other industrial applications due to their excellent flame retardancy and plasticizing properties [1]. China is the world’s largest producer, consumer, and exporter of CPs [2]. The global CPs market size is expected to rise to US$21 billion by 2030, with a compound annual growth rate of 5.6%, while China dominates the market with over 40% of global production capacity [3]. Based on carbon chain length, CPs are categorized into short chain CPs (SCCPs, C_10–13_), medium chain CPs (MCCPs, C_14–17_), and long chain CPs (LCCPs, C_18–30_). It is noteworthy that SCCPs and MCCPs have been listed under the Stockholm Convention for global regulation due to their toxicity, persistence, long-range transport potential, and bioaccumulative properties [4]. Accordingly, China implemented a comprehensive phase-out of SCCPs effective 1 January 2024; the European Union, Canada, and other countries have restricted their production and use, with MCCPs being listed as candidates and gradually incorporated into regulatory frameworks [5,6]. However, current toxicological data on CPs remain limited, hindering accurate assessment of their potential ecological risks.

Upon release into the environment, CPs tend to accumulate in aquatic systems, which serve as important sinks [7]. Wu et al. (2021) reported SCCPs concentrations as high as 70.3 μg/L in groundwater surrounding CPs production facilities [8]. Surface water in China’s Baiyangdian Lake and the Yangtze River basin has been reported to contain SCCPs in the ranges of 1563–56,306 ng/L and 1131–65,640 ng/L, respectively [9]. Substantial evidence indicates that the ecotoxicological effects of CPs are both species- and concentration-dependent. For instance, Peng et al. (2019) demonstrated that exposure to low doses (<100 ppb) of SCCPs induced genetic damage in zebrafish and disrupted energy metabolism and metal ion binding pathways [10]. In addition, Chen et al. (2024) reported that MCCPs exposure primarily perturbed lipid metabolism as well as amino acid and nucleotide metabolism in zebrafish larvae [11]. Studies have shown that the sensitivity of different aquatic organisms to SCCPs followed the order: *Daphnia magna* > *Brachionus calyciflorus* > fish > *Chironomus tentans* > algae. Among the algal species tested, *Chlorella pyrenoidosa* was the most sensitive while *Scenedesmus quadricauda* was the least sensitive, with EC_50_ values of 168 μg/L and 1086 μg/L, respectively [12]. Although previous studies have preliminarily revealed the toxic effects of CPs on individual aquatic organisms such as zebrafish [6,7,9], direct comparative evidence regarding the differential toxicity of CPs with varying carbon chain lengths between the two major groups of primary producers in aquatic ecosystems, specifically eukaryotic microalgae and prokaryotic microalgae, remains lacking. Numerous studies have demonstrated that eukaryotic and prokaryotic microalgae exhibit fundamental differences in metabolic remodeling and regulatory responses under pollutant stress [13,14,15,16]. Furthermore, existing toxicological evidence has largely focused on phenotypic endpoints, while the molecular mechanisms underlying how carbon chain length drives selective toxicity remain poorly understood.

Based on this, the present study selected the representative eukaryotic algae *Chlorella* sp. and the prokaryotic algae *Microcystis aeruginosa* (*M. aeruginosa*) as test organisms to evaluate the differential toxic effects of CPs with different carbon chain lengths on algal growth, photosynthetic pigment, oxidative stress, cell morphology, and molecular-level responses. The study further explores the mechanistic basis for the different toxic responses of microalgae with distinct structural types to CPs of different carbon chain lengths. This study fills a critical gap in the comparative toxicology of CPs between eukaryotic algae Chlorella sp. and the prokaryotic algae *M. aeruginosa*, and provides a scientific basis for assessing the potential hazards of CPs to primary producers in aquatic ecosystems.

## 2. Materials and Methods

### 2.1. Exposure Experiment

The eukaryotic algae *Chlorella* sp. (FACHB-5) and prokaryotic algae *Microcystis aeruginosa* Kützing (FACHB-315) were obtained from the Freshwater Algae Culture Collection at the Institute of Hydrobiology (Wuhan, China, http://algae.ihb.ac.cn/). Standards for SCCPs (C_10_–C_13_, 51.5% Cl), MCCPs (C_14_–C_17_, 52% Cl), and LCCPs (C_18_–C_20_, 49% Cl) were purchased from Dr. Ehrenstorfer GmbH (Augsburg, Germany). Based on environmental concentrations of CPs and relevant exposure studies [8,9,12,17,18,19] (Table S1), concentration gradients of 0.01 mg/L, 0.1 mg/L, and 1.0 mg/L were established for this study, with experimental groups detailed in Table S2. Dimethyl sulfoxide (DMSO) was used as a solvent aid, with its final concentration not exceeding 0.1% in all experimental solutions. The initial algal densities of *Chlorella* sp. and *M. aeruginosa* ranged from 5.5 × 10^6^ to 5.8 × 10^6^ cells/mL. Algal cells were grown in BG11 medium at 25 ± 1 °C with a 12 h light:12 h dark photoperiod. The cultures were placed in an illuminated incubator (GDN-1000D-4, Ningbo Southeast Instrument Co., Ltd., Ningbo, China) with a light intensity of 3000 ± 50 lux. The cultures were kept in 500 mL conical flasks, each containing 200 mL of algal suspension. The flasks were maintained under static conditions and manually shaken 3 times daily to ensure uniform cell suspension and adequate gas exchange. The experiment lasted for 96 h, and each treatment group was conducted in triplicate.

### 2.2. Determination of Physiological and Biochemical Indicators

Algal densities of *Chlorella* sp. and *M. aeruginosa* were measured at 48 h and 96 h using an algal cell counter (Countstar^®^ IM 1000, Shanghai Ruiyu Biotech Co., Ltd., Shanghai, China), and the growth inhibition rate was calculated (Text S1). After the experiment, 5.0 mL of algal suspension was centrifuged using a refrigerated centrifuge (3K15, Sigma-Aldrich (Shanghai) Co., Ltd., Shanghai, China) at 6000 rpm for 15 min at 4 °C. The supernatant was discarded, and 90% acetone was added for pigment extraction under dark conditions at 4 °C for 24 h. Following extraction, the sample was centrifuged again at 6000 rpm for 10 min to obtain the supernatant. Using 90% acetone as a blank reference, the absorbance of the supernatant was measured at wavelengths of 664 nm and 647 nm, and the chlorophyll-a (Chl-a) content was calculated [20]. More details can be found in Text S2. At the end of the experiment, 50.0 mL of algal suspension was centrifuged at 3000 rpm for 10 min at 4 °C. The supernatant was discarded, and the cells were resuspended in phosphate-buffered saline (PBS). The samples were homogenized using a high-throughput tissue grinder for 5 min at 4 °C, repeated three times. Subsequently, the samples were centrifuged at 6000 rpm for 10 min at 4 °C, and the supernatant was retained. The activities of superoxide dismutase (SOD), catalase (CAT), glutathione S-transferase (GST) and peroxidase (POD), as well as the contents of soluble protein, malondialdehyde (MDA) and glutathione (GSH), were measured according to the protocol of the assay kits (Suzhou Coming Biotechnology Co., Ltd., Suzhou, China) using a microplate reader (CyTation3, Biotek Instruments, Winooski, VT, USA). Additionally, 10.0 mL of algal culture was collected and centrifuged at 8000 rpm for 10 min at 4 °C. The supernatant was collected, and the extracellular contents of Microcystin-LR (MC-LR) and Microcystin-RR (MC-RR) from *M. aeruginosa* were determined using MC-LR and MC-RR ELISA kits (Changdaen Biotechnology (Shanghai) Co., Ltd., Shanghai, China).

### 2.3. Comparative Analysis of Comprehensive Stress Resistance of Algae

This study employed the membership function method to conduct a comprehensive evaluation of the physiological and biochemical indicators of *Chlorella* sp. and *M. aeruginosa* across different treatment groups (Text S4). The aim was to compare the comprehensive stress resistance of the eukaryotic (*Chlorella* sp.) and prokaryotic (*M. aeruginosa*) algae upon exposure to CPs of different carbon chain lengths [21].

### 2.4. Analysis of Cellular Morphology and Ultrastructure

The 30.0 mL of algal suspension was centrifuged at 2000× *g* for 10 min at 4 °C to collect algal cells. The supernatant was discarded, and the cells were fixed with 2.5% glutaraldehyde solution overnight in the dark at 4 °C. The pre-treated samples were subsequently observed using scanning electron microscopy (SEM, Hitachi Model TM-1000, Tokyo, Japan) and transmission electron microscopy (TEM, Hitachi H-7650, Tokyo, Japan). Detailed pre-treatment procedures are provided in Text S3. For each sample, a minimum of ten fields of view were examined to ensure that the presented images were representative of the predominant damage patterns observed.

### 2.5. Transcriptomic Analysis

50.0 mL algal suspension was centrifuged at 3500 rpm for 5 min at 4 °C for enrichment. The pellet was resuspended in PBS buffer and stored at −80 °C. RNA sequencing was performed by Majorbio Bio-pharm Technology Co., Ltd. (Shanghai, China). Detailed information is provided in Text S5. Differential expression analysis was performed using the DESeq2 software (version 1.48.2), with the screening criteria for differentially expressed genes (DEGs) set at |Log_2_FoldChange| > 1 and adjusted *p*-value < 0.05. Gene Ontology (GO) and Kyoto Encyclopedia of Genes and Genomes (KEGG) analyses were utilized for the functional annotation of the differentially expressed genes.

### 2.6. Statistics Analysis

Data are presented as the mean ± standard deviation (*n* = 3). Statistical analyses were performed using SPSS software (IBM SPSS Statistics, version 26.0). One-way analysis of variance (ANOVA) was conducted to assess overall differences among groups, followed by Duncan’s new multiple range test for post hoc multiple comparisons. Differences were considered statistically significant at *p* < 0.05. The bioinformatics analyses were performed using the online platform of Majorbio Cloud Platform (https://cloud.majorbio.com/) from Shanghai Majorbio Bio-pharm Technology Co., Ltd. (Shanghai, China).

## 3. Results and Discussion

### 3.1. Effects of CPs on the Growth and Photosynthetic Pigments in Chlorella *sp.* and M. aeruginosa

**Figure 1:**
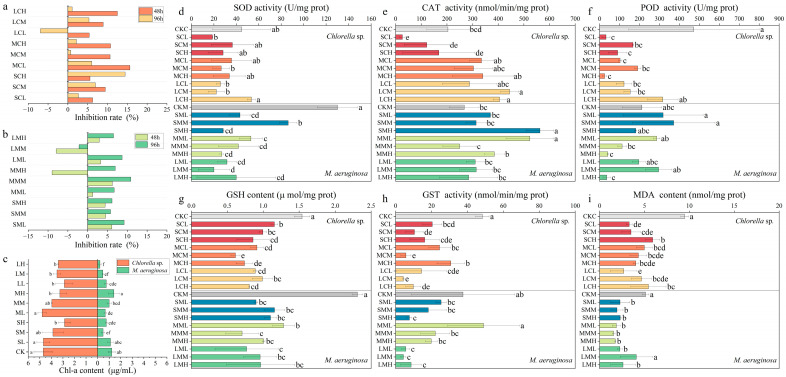
Effects of SCCPs, MCCPs, and LCCPs treatment with different concentrations on growth (**a**,**b**), Chl-a content (**c**), and antioxidant capacity (**d**–**i**) of *Chlorella* sp. and *M. aeruginosa.* (**a**) growth inhibition of *Chlorella* sp. (**b**) growth inhibition of *M. aeruginosa.* (**c**) Chl-a content. (**d**) SOD activity. (**e**) CAT activity. (**f**) POD activity. (**g**) GSH content. (**h**) GST activity. (**i**) MDA content. “CKC” represents the control group for *Chlorella* sp., and “CKM” represents the control group for *M. aeruginosa*. For the treatment groups, the abbreviations follow a consistent three-part structure: the first letter denotes the type of pollutant (S for SCCPs, M for MCCPs, L for LCCPs), the second letter denotes the algal species (C for *Chlorella* sp., M for *M. aeruginosa*), and the third letter denotes the concentration level (L for 0.01 mg/L, M for 0.1 mg/L, H for 1.0 mg/L). Detailed information is provided in Table S2. Different lowercase letters indicate significant differences (*p* < 0.05).

The growth inhibition rate of microalgae is a direct indicator of the toxicity of CPs [22]. In this study, CPs with different carbon chain lengths were found to significantly inhibit the growth of both the eukaryotic algae *Chlorella* sp. and the prokaryotic algae *M. aeruginosa* (Figure 1a,b). Similar cytotoxicity in HepG2 cells induced by CPs of different chain lengths has been reported; the average cell viability following SCCPs exposure was slightly lower than that observed in the MCCPs and LCCPs treated groups [23]. Exposure to SCCPs at 0.01 to 1.0 mg/L inhibited the growth of both *Chlorella* sp. and *M. aeruginosa* in this study. Following exposure to CPs of different carbon chain lengths, *Chlorella* sp. exhibited pronounced growth inhibition at 48 h. At 96 h, all treatments caused growth inhibition except for the 0.01 mg/L LCCPs treatment. Specifically, the growth inhibition rate of *Chlorella* sp. by SCCPs showed a positive correlation with concentration, reaching a maximum of 14.45%. For *M. aeruginosa*, exposure to 1.0 mg/L MCCPs for 48 h produced a significant stimulatory effect, with growth increased by up to 8.98%. Furthermore, as exposure duration increased from 48 h to 96 h, the toxicity of CPs to *M. aeruginosa* also increased. The inhibitory effect of CPs of different carbon chain lengths on Chl-a content in *Chlorella* sp. and *M. aeruginosa* followed the order: LCCPs > SCCPs > MCCPs (Figure 1c). SCCPs and MCCPs treatments produced concentration-dependent inhibition of Chl-a content in *Chlorella* sp. Compared with the control group, exposure to 1.0 mg/L SCCPs and 1.0 mg/L MCCPs significantly decreased Chl-a content (*p* < 0.05). All tested concentrations of LCCPs significantly reduced Chl-a content in *Chlorella* sp. (*p* < 0.05). The distinct sensitivity patterns observed between the two algal species can be attributed to fundamental differences in their cellular architecture and associated physiological mechanisms. As a eukaryotic algae, *Chlorella* sp. possesses a rigid cell wall composed of cellulose and glycoproteins, which serves as a physical barrier against xenobiotic penetration, along with membrane-bound organelles such as chloroplasts, mitochondria, and vacuoles that enable compartmentalization of metabolic processes and sequestration of toxicants [24]. In contrast, the prokaryotic algae *M. aeruginosa* lacks a true cell wall and membrane-bound organelles; its cell surface is characterized by a more negatively charged peptidoglycan layer with distinct functional group composition, which may facilitate direct interactions with environmental pollutants [16,24]. Furthermore, differences in photosynthetic apparatus organization may contribute to differential susceptibility to photosystem-targeting stressors. In *Chlorella* sp., the photosystems are structurally protected within the chloroplasts, whereas in *M. aeruginosa*, the photosystems are embedded in the thylakoid membranes [16,24,25]. Additionally, the antioxidant defense capacity and the ability to activate stress-responsive transcriptional programs have been shown to vary substantially between eukaryotic and prokaryotic algae, collectively shaping their distinct adaptive responses to chemical exposure [16,25].

### 3.2. Effects of CPs on Oxidative Damage in Chlorella *sp.* and M. aeruginosa

**Figure 2:**
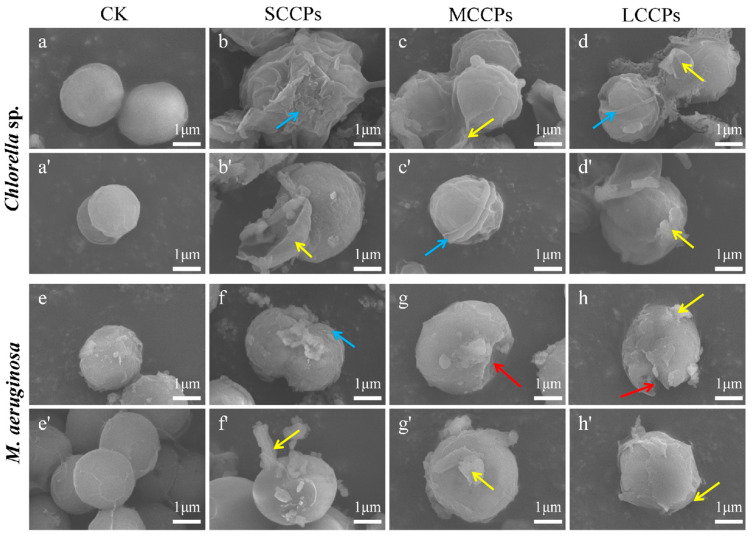
SEM images of *Chlorella* sp. and *M. aeruginosa* exposed to CPs with different carbon chain lengths. (**a**,**a′**) *Chlorella* sp., control; (**b**,**b′**) *Chlorella* sp., exposed to SCCPs; (**c**,**c′**) *Chlorella* sp., exposed to MCCPs; (**d**,**d′**) *Chlorella* sp., exposed to LCCPs; (**e**,**e′**) *M. aeruginosa*, control; (**f**,**f′**) *M. aeruginosa*, exposed to SCCPs; (**g**,**g′**) *M. aeruginosa*, exposed to MCCPs; (**h**,**h′**) *M. aeruginosa*, exposed to LCCPs. For each treatment group, a minimum of ten fields of view were examined, and representative images were selected to illustrate characteristic morphological changes observed. Blue arrow, cell surface shrinkage; red arrow, cell surface holes; yellow arrow, shed surface plates.

**Figure 3:**
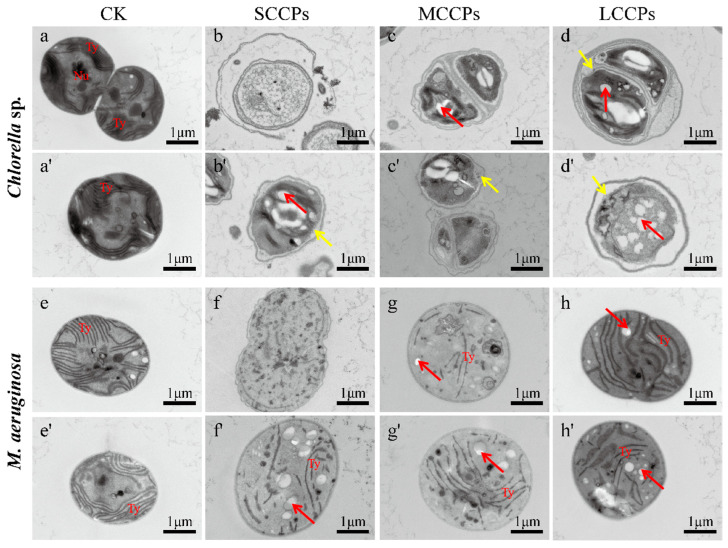
TEM images of *Chlorella* sp. and *M. aeruginosa* exposed to CPs with different carbon chain lengths. (**a**,**a′**) *Chlorella* sp., control; (**b**,**b′**) *Chlorella* sp., exposed to SCCPs; (**c**,**c′**) *Chlorella* sp., exposed to MCCPs; (**d**,**d′**) *Chlorella* sp., exposed to LCCPs; (**e**,**e′**) *M. aeruginosa*, control; (**f**,**f′**) *M. aeruginosa*, exposed to SCCPs; (**g**,**g′**) *M. aeruginosa*, exposed to MCCPs; (**h**,**h′**) *M. aeruginosa*, exposed to LCCPs. For each treatment group, a minimum of ten fields of view were examined, and representative images were selected to show typical ultrastructural alterations observed. Thylakoid, Ty; Nucleus, Nu; yellow arrow, plasmolysis; red arrow, cytoplasmic vacuolization.

Exposure to eorganic pollutants induces antioxidant defense mechanisms in microalgae to maintain intracellular homeostasis [26,27]. In this study, the antioxidant responses of the eukaryotic alga *Chlorella* sp. and the prokaryotic algae *M. aeruginosa* exhibited pronounced differences under exposure to CPs with different carbon-chain lengths (Figure 1d–i). SCCPs and MCCPs generally suppressed SOD activity in both algal species. In contrast, exposure of *Chlorella* sp. to 1.0 mg/L LCCPs resulted in a significantly higher SOD activity compared to other LCCPs concentration groups (*p* < 0.05), indicating that higher concentrations of LCCPs more strongly induce oxidative stress. Conversely, the SOD activity in *M. aeruginosa* was significantly inhibited across treatments with CPs with different carbon chain lengths compared to the control group (*p* < 0.05). In *Chlorella* sp., exposure to MCCPs and LCCPs generally induced an increase in CAT activity, whereas exposure to 0.01 mg/L SCCPs produced a significant inhibition (*p* < 0.05). These results indicated that when CPs carbon chain length or exposure concentration exceeds a certain threshold, *Chlorella* sp. tended to enhance CAT activity to eliminate accumulated H_2_O_2_. Regarding *M. aeruginosa*, CAT activity was significantly higher than in the control group upon exposure to 1.0 mg/L SCCPs and 0.01 mg/L MCCPs (*p* < 0.05). After exposure to CPs of different chain lengths and concentrations, the POD activity in *Chlorella* sp. tended to be suppressed overall, suggesting an impaired capacity for H_2_O_2_ scavenging or damage to the antioxidant defense system. In contrast, exposure to CPs more readily enhanced POD activity in *M. aeruginosa*, which facilitated the scavenging of H_2_O_2_ generated by CPs-induced stress. The GSH content in both *Chlorella* sp. and *M. aeruginosa* exposed to CPs of different carbon chain lengths was significantly lower than that of the control group (*p* < 0.05). Under exposure to 0.01 mg/L MCCPs, *M. aeruginosa* showed increased GST activity accompanied by a relatively higher GSH content (*p* < 0.05). Notably, MDA content in both species under exposure to CPs of varying carbon chain lengths was lower than in control groups. Moreover, we observed that the synthesis and release of extracellular microcystins MC-LR and MC-RR by *M. aeruginosa* were influenced by both the carbon chain length and the concentration of CPs (Figure S1). The extracellular MC-LR content was higher than that in the control group, whereas MC-RR exhibited the opposite pattern. Specifically, extracellular MC-LR content under 0.1 mg/L LCCPs was significantly greater than in the control group, while extracellular MC-RR content under 1.0 mg/L SCCPs was significantly lower than in the control (*p* < 0.05). Notably, SEM and TEM observations (Figure 2 and Figure 3) revealed that CPs exposure caused evident morphological damage to *M. aeruginosa* cells, including surface shrinkage, membrane deformation, and disruption of cellular integrity. These structural alterations suggested that CPs may compromise cell membrane integrity, which could potentially influence the release of intracellular metabolites such as microcystins. Previous studies reported that *M. aeruginosa* can increase MC-LR production in response to H_2_O_2_ stress, as an adaptive response [28]. Zhang et al. (2024) also demonstrated that exposure to two widely used quaternary ammonium compound salts, benzalkonium chloride and benzalkonium bromide, promoted the synthesis and release of MC-LR in *M. aeruginosa* [29].

Based on the above physiological and biochemical indicators, we compared the comprehensive stress resistance of the two structurally distinct algae, eukaryotic algae *Chlorella* sp. and prokaryotic algae *M. aeruginosa*, following exposure to CPs with different carbon chain lengths (Table S3). The comprehensive stress resistance index for *Chlorella* sp. was 3.125, slightly higher than that for *M. aeruginosa* (3.088), indicating that *Chlorella* sp. Exhibited greater tolerance to CPs (Table S3). Previous studies have shown that exposure to sodium hypochlorite (NaClO) at concentrations far below 0.01 mg/L inhibited the photosynthetic activity of both *Chlorella vulgaris* and *M. aeruginosa*. In *M. aeruginosa*, the esterase activity and ROS concentration were strongly correlated with NaClO exposure, indicating that *M. aeruginosa* is more sensitive to NaClO than *Chlorella vulgaris* [15]. In this study, both *Chlorella* sp. and *M. aeruginosa* exhibited their highest stress resistance to MCCPs. The ranking of comprehensive stress resistance for *Chlorella* sp. from highest to lowest was: MCCPs (3.496) > SCCPs (3.305) > LCCPs (2.574). For *M. aeruginosa*, the order was: MCCPs (3.148) > LCCPs (3.085) > SCCPs (3.030).

### 3.3. Effects of CPs on the Morphological and Ultrastructural Properties of Chlorella *sp.* and M. aeruginosa

The morphological characteristics of the eukaryotic algae *Chlorella* sp. and the prokaryotic algae *M. aeruginosa* cells following exposure to CPs were observed using SEM (Figure 2). Cells in the control groups of both *Chlorella* sp. and *M. aeruginosa* exhibited a spherical shape with smooth surfaces and remained structurally intact. By contrast, cells exposed to CPs of different carbon chain lengths exhibited various deformative features, including surface shrinkage (blue arrow), holes (red arrow), and even shedding of surface plates (yellow arrow). As can be seen from Figure 2, CPs with the same carbon chain length inflicted more severe physical damage to *Chlorella* sp. Numerous studies have shown that exposure to pollutants can induce mechanical damage in microalgae, including cell wall impairment and cell fragmentation, thereby compromising the structural integrity of algal cells [16,30]. In the present study, the formation of the observed surface holes may be attributed to the mechanical damage induced by CPs. Under exposure to MCCPs, the cellular structures of both *Chlorella* sp. and *M. aeruginosa* remained relatively more intact compared to those exposed to SCCPs and LCCPs. Nevertheless, surface morphology was altered, with shedding of the outer cell layer and occasional deposition of flocculent material on the cell surface.

Furthermore, TEM results revealed that CPs induced significant ultrastructural damage in both the eukaryotic algae *Chlorella* sp. and the prokaryotic algae *M. aeruginosa*, with the extent of damage varying depending on the carbon chain length and algal species (Figure 3). In the control groups of both algae, the internal cell structures were stable, organelles remained intact, and the thylakoid lamellar structures were clearly visible and arranged in an approximately parallel manner. Under exposure to CPs of the same carbon chain length, the damage to the internal cellular structure of *Chlorella* sp. was more severe than that observed in *M. aeruginosa*. However, exposure to CPs of different carbon chain lengths produced disruption or curling of the thylakoid lamellae, increased numbers of electron dense bodies, and cytoplasmic vacuolization, indicating that CPs exposure compromised membrane structures and photosynthetic organelles in both species. Damage to algal organelles can impair growth and metabolism, and, in particular, thylakoid disruption can lead to inhibition of Chl-a content and even induce oxidative stress. Consequently, the structural damage inflicted by CPs exposure inhibited the growth and photosynthetic activity observed in this study (Figure 1). After 96 h of exposure to CPs with different carbon chain lengths, *Chlorella* sp. displayed severe intracellular damage characterized by numerous vacuoles of varying sizes (red arrow), partial depolymerization and dispersion of thylakoid, and a generally blurred cellular outline. In addition, the plasma membrane detached from the cell wall, and in some cells, the cell membrane was deformed or ruptured (yellow arrow). In the SCCPs’ treatment, cytoplasmic leakage accompanied by the release of abundant electron-dense granules was observed. In contrast, *M. aeruginosa* cells exposed to the same concentration of SCCPs (1.0 mg/L) did not exhibit rupture. Comprehensive analysis indicates that the intracellular structural damage in *Chlorella* sp. was more severe than that in *M. aeruginosa.*

These observations collectively indicate that CPs compromise the integrity of the cell membrane and cell wall. Studies have shown that polychlorinated biphenyls (PCBs) partition into lipid bilayers and subsequently accumulate in internal membranes such as thylakoids [31]. The physical disruption of membrane structure observed in our study, including deformation and rupture, likely further facilitates the entry of CPs into the cytoplasm. Previous research has demonstrated that CPs exhibit high bioaccumulation potential, with log bioconcentration factors reaching 6.7 to 7.0 in *Daphnia magna* [32]. Notably, CPs congeners with higher octanol-water partition coefficients show preferential accumulation in lipid-rich tissues such as the liver [33], which is consistent with the hydrophobic partitioning behavior of CPs. Together, these findings support our morphological observations that the disruption of membrane integrity provides a physical pathway for CPs to enter cells. Once internalized, CPs are likely to partition into thylakoid membranes, consistent with the ultrastructural damage observed by TEM.

### 3.4. Transcriptional Response of Chlorella *sp.* and M. aeruginosa to CPs

#### 3.4.1. Differentially Expressed Genes

**Figure 4:**
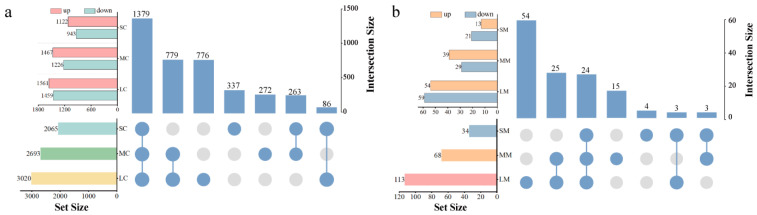
Effects of exposure to CPs with different carbon chain lengths on gene expression in *Chlorella* sp. (**a**) and *M. aeruginosa* (**b**). Blue circles indicate that the treatment group is included in the corresponding column combination; gray circles indicate exclusion.

To elucidate the potential mechanisms underlying the observed toxic effects, transcriptomic analyses were conducted on the eukaryotic algae *Chlorella* sp. and the prokaryotic algae *M. aeruginosa* exposed to 1.0 mg/L CPs with different carbon chain lengths for 96 h. The results showed that CPs’ exposure significantly altered gene regulation in both algal species. The influence of LCCPs on gene regulation was more pronounced than that of MCCPs and SCCPs in both algae. In *Chlorella* sp., the number of upregulated DEGs exceeded that of downregulated DEGs following exposure to CPs with different carbon chain lengths, indicating that CPs tended to promote gene expression. In contrast, for *M. aeruginosa*, SCCPs and LCCPs exposure predominantly promoted gene expression, whereas MCCPs exposure tended to suppress it (Figure 4).

#### 3.4.2. GO Enrichment Analysis

**Figure 5:**
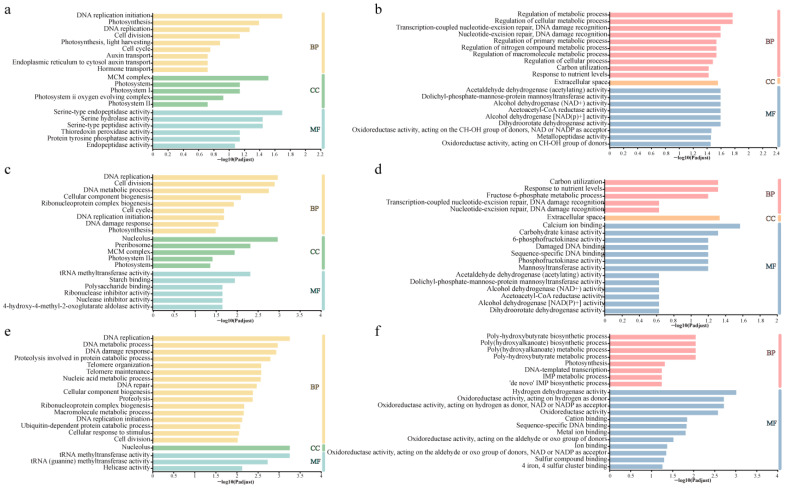
GO enrichment analysis of *Chlorella* sp. and *M. aeruginosa* after exposure to CPs with different carbon chain lengths. (**a**,**c**,**e**) GO enrichment in *Chlorella* sp. under SCCPs, MCCPs, and LCCPs exposure, respectively. (**b**,**d**,**f**) GO enrichment in *M. aeruginosa* under SCCPs, MCCPs, and LCCPs exposure, respectively. BP, Biological Process; CC, Cellular Component; MF, Molecular Function.

To illustrate the specific biological functions of DEGs in the eukaryotic algae *Chlorella* sp. and the prokaryotic algae *M. aeruginosa* exposed to CPs with different carbon chain lengths (SCCPs vs. CK, MCCPs vs. CK, and LCCPs vs. CK), GO enrichment analysis was performed. The top 20 most significantly enriched GO terms in the categories molecular function, cellular component, and biological process are shown in Figure 5.

The GO enrichment analysis results showed that *Chlorella* sp. exposed to SCCPs exhibited the highest number of significantly enriched terms in the biological process category. Among these, the pathway with the largest number of enriched DEGs was DNA replication initiation. As a critical step for cell proliferation and growth, the regulation of DNA replication initiation not only determines cell cycle progression but also indirectly modulates the expression of photosynthesis-related and metabolic genes by influencing the timing of genome replication [34]. In the molecular function category, serine-type endopeptidase activity was the most enriched GO term. Serine endopeptidases play central roles in protein degradation, modification, and transport, thereby affecting growth, development, and defense processes [35]. Regarding cellular components, the MCM complex was enriched with the highest number of DEGs. The MCM complex is responsible for unwinding the DNA double helix during replication, ensuring proper synthesis of new strands and maintaining growth and genetic stability [36]. Notably, GO enrichment patterns for *Chlorella* sp. under MCCPs and LCCPs exposure were similar. In both treatment groups, significantly enriched terms included DNA replication in biological process, tRNA methyltransferase activity in molecular function, and nucleolus in cellular component, indicating that MCCPs and LCCPs primarily affect DNA replication, ribosome biogenesis, and translation processes in *Chlorella* sp.

For *M. aeruginosa*, exposure to SCCPs significantly affected the regulation of metabolic processes and acetaldehyde dehydrogenase (acetylating) activity. The upregulation of acetaldehyde dehydrogenase (ALDH) is generally regarded as a molecular marker of stress response, serving a dual role in detoxifying aldehydes, reducing oxidative stress, and generating reduced nicotinamide adenine dinucleotide (NADH) to support antioxidant defense systems [37]. Therefore, the induction of ALDH likely enhances cellular antioxidant capacity both by directly scavenging toxic aldehydes and by providing NADH, potentially leading to the indirect upregulation of antioxidant enzymes such as SOD and CAT (Figure 1). Under MCCPs’ exposure, the most significantly enriched terms in *M. aeruginosa* were response to carbon utilization in the biological process category and calcium ion binding in the molecular function category. In response to pollutant stress, algae frequently modulate carbon metabolic pathways or reallocate carbon resources to mitigate toxicity and preserve energy balance [38]. Calcium, as a key signaling and structural element, participates in cell division, elongation, and programmed cell death, and modulates antioxidant defenses, thereby playing a key role in enhancing tolerance to oxidative stress [39]. In the LCCPs treatment group, *M. aeruginosa* was significantly enriched in terms related to the poly-hydroxybutyrate (PHB) biosynthetic process and hydrogen dehydrogenase activity. Excessive PHB synthesis can impose a metabolic burden on cells, diverting carbon and energy resources away from growth and photosynthesis, ultimately inhibiting growth and photosynthetic efficiency [40]. No significantly enriched pathways were identified in the cellular component category for the LCCPs treatment. In contrast, both the SCCPs and MCCPs treatments showed the greatest DEGs enrichment in the extracellular space. The structure and composition of the extracellular compartment influence how algae store and utilize nutrients and ions, and thus indirectly affect cellular metabolism and physiology [41]. This suggests that SCCPs and MCCPs exposure may alter the extracellular matrix and secreted material, modifying exchanges between the cell and its environment and affecting defensive responses. The GO enrichment characteristics of the two algae under CPs exposure were directly consistent with the phenotypic changes in growth inhibition and photosynthetic efficiency reduction at the physiological level, and also correlated with the changes in oxidative stress-related indicators detected by biochemical assays at the molecular level. The differences in GO enrichment induced by CPs with different carbon chain lengths.

#### 3.4.3. KEGG Enrichment Analysis

**Figure 6:**
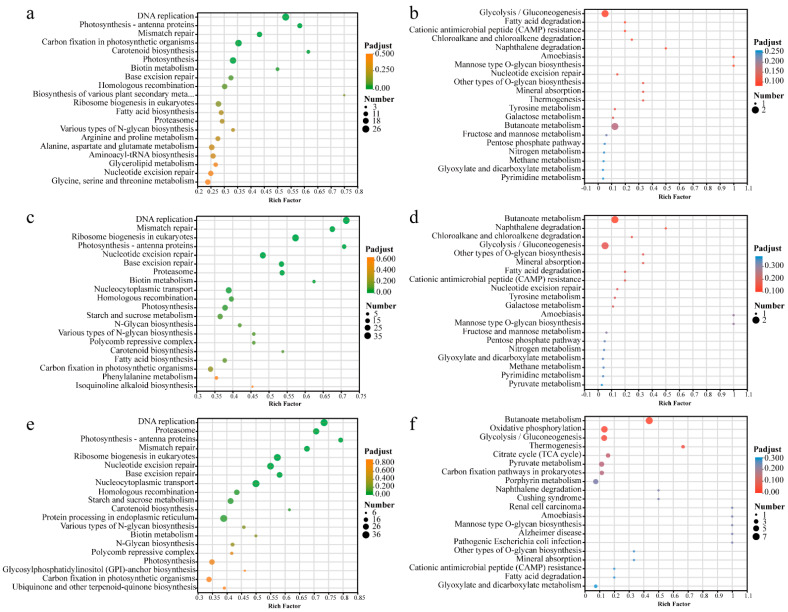
KEGG enrichment analysis of *Chlorella* sp. and *M. aeruginosa* after exposure to CPs with different carbon chain lengths. (**a**,**c**,**e**) KEGG enrichment in *Chlorella* sp. under SCCPs, MCCPs, and LCCPs exposure, respectively. (**b**,**d**,**f**) KEGG enrichment in *M. aeruginosa* under SCCPs, MCCPs, and LCCPs exposure, respectively.

To validate the biological pathways activated in the eukaryotic algae *Chlorella* sp. and the prokaryotic algae *M. aeruginosa* following exposure to CPs with different carbon chain lengths, we performed KEGG pathway enrichment analysis (Figure 6). In *Chlorella* sp., exposure to CPs significantly suppressed pathways related to DNA replication and mismatch repair pathways, indicating that CP-induced stress adversely affects the preparatory stages of cell proliferation and genomic stability. In addition, SCCPs’ exposure significantly upregulated pathways for photosynthesis: antenna proteins and carbon fixation are present in photosynthetic organisms, suggesting that SCCPs exert pronounced regulatory effects on energy metabolism and DNA replication. Notably, mixotrophic *Ochromonas gloeopara* grazing on toxic *M. aeruginosa* has been reported to significantly upregulate genes involved in photosynthesis and carbon fixation [42]. Under MCCPs’ exposure, *Chlorella* sp. showed significant enrichment in ribosome biogenesis in eukaryotes. The downregulation of this pathway is known to directly impair cellular growth rates and protein synthesis capacity [43]. In the LCCPs treatment, the proteasome pathway was significantly enriched. The proteasome is essential for degrading cyclins and regulatory factors, a process essential for maintaining cell cycle progression and adapting to environmental perturbations [44]. Its downregulation may result in the accumulation of damaged or misfolded proteins and disruption of proteostasis.

KEGG pathway enrichment analysis of DEGs in *M. aeruginosa* exposed to CPs of different carbon chain lengths revealed pronounced chain length-dependent responses in pathways related to energy metabolism, carbon metabolic reprogramming, and oxidative stress, and these pathways were significantly downregulated in the CPs treatment groups. Under SCCP exposure, genes were significantly enriched in glycolysis/gluconeogenesis. As a central pathway of cellular energy metabolism, its disruption directly reduces ATP production and impairs microalgal growth and physiological activities. The butanoate metabolism pathway was significantly enriched in the SCCPs, MCCPs, and LCCPs treatment groups. Butanoate could be metabolized by β-oxidation to generate acetyl-CoA, which then enters the citric acid cycle to supply energy for various cellular processes [45]. The downregulation of butanoate metabolism indicated that SCCPs, MCCPs, and LCCPs induce disruption of energy-yielding pathways. Both the SCCPs and MCCPs treatments showed downregulation of naphthalene degradation, and the MCCPs treatment additionally exhibited downregulation of chloroalkane and chloroalkene degradation pathways. In the LCCPs treatment, the oxidative phosphorylation pathway was significantly enriched and markedly downregulated, indicating further impairment of energy metabolism in *M. aeruginosa*. The KEGG pathway enrichment results under CPs exposure clearly explained the molecular mechanism of abnormal physiological and biochemical indicators, and the down-regulation of pathways related to energy metabolism and DNA replication was highly consistent with the growth inhibition.

### 3.5. Toxicity Mechanisms of SCCPs, MCCPs, and LCCPs on Chlorella *sp.* and M. aeruginosa

**Figure 7:**
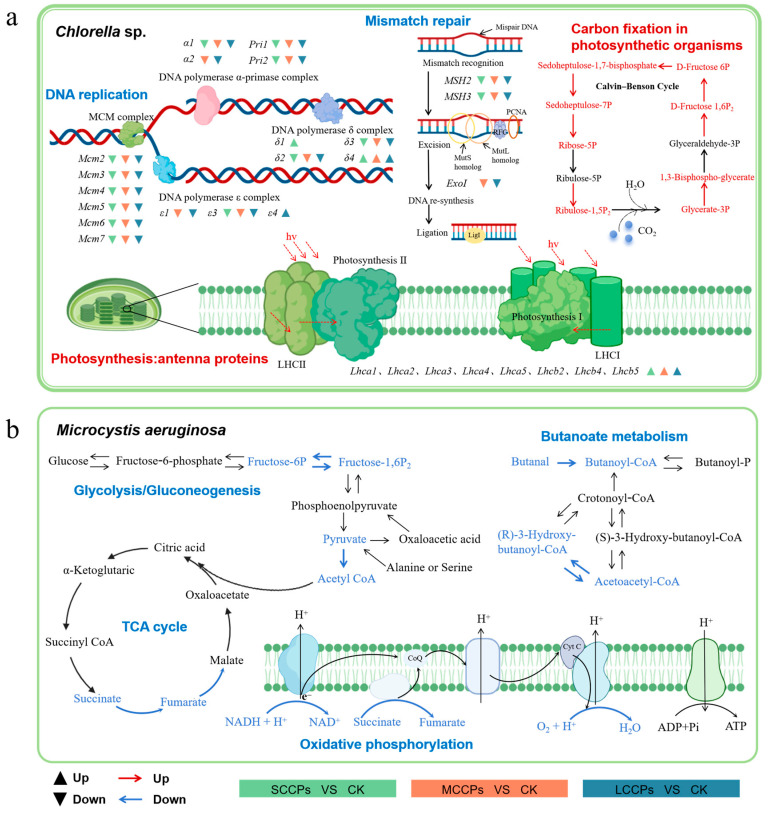
Potential toxicity mechanisms of CPs with different carbon chain lengths against *Chlorella* sp. (**a**) and *M. aeruginosa* (**b**). The toxicity mechanisms are proposed based on the integrated analysis of differentially expressed genes and KEGG pathway enrichment. Arrows and connections represent hypothesized regulatory relationships derived from the transcriptomic data.

We found that CPs with different carbon chain lengths significantly affected the DNA replication and photosynthesis pathways in the eukaryotic algae *Chlorella* sp. (Figure 7a). Exposure to SCCPs, MCCPs, and LCCPs significantly suppressed the expression of genes associated with DNA replication and mismatch repair (*p* < 0.05). During the algal life cycle, the precise coordination between DNA replication and mismatch repair is fundamental to the high-fidelity transmission of genetic information. Our results indicated that exposure to CPs with different chain lengths led to a general downregulation of DNA replication-related genes in *Chlorella* sp., suggesting impairments in replication initiation and replication fork progression. Specifically, downregulation was observed in genes encoding subunits of the minichromosome maintenance (MCM) complex, including *Mcm2*, *Mcm3*, *Mcm4*, *Mcm5*, *Mcm6*, and *Mcm7*, which are essential for replication initiation. Genes encoding the DNA polymerase α-primase complex (*α1*, *Pri1*, and *Pri2*) were downregulated, and genes encoding the DNA polymerase δ complex (δ2 and δ3) were also downregulated. In the mismatch repair pathway, the key genes *MSH2* and *MSH3* were significantly downregulated in all treatment groups, while the exonuclease I-encoding gene *ExoI* showed downregulation in the MCCPs and LCCPs treatment groups. The concomitant impairment of DNA replication and mismatch repair mechanisms may compromise genomic stability and increase the risk of replication errors being retained. Photosynthesis is indispensable in algal growth, providing not only the energetic foundation but also facilitating carbon fixation essential for biomass synthesis and biogeochemical cycling. Exposure to CPs with different carbon chain lengths induced upregulation of genes encoding light-harvesting complex proteins, which are responsible for light capture and energy transfer. Specifically, enriched genes included *Lhca1*, *Lhca2*, *Lhca3*, *Lhca4*, *Lhca5*, *Lhcb2*, *Lhcb4*, and *Lhcb5*, indicating that the algal cells enhance light absorption and energy transfer as a compensatory or adaptive response under CP’s stress. Furthermore, the pathway of carbon fixation in photosynthetic organisms was globally upregulated, suggesting that *Chlorella* sp. can enhance photosynthetic carbon assimilation efficiency under SCCP stress, thereby supporting the energy and biosynthetic precursors required for emergency metabolism and damage repair.

CPs’ exposure significantly perturbed key biological pathways in the prokaryotic algae *M. aeruginosa*, including butanoate metabolism, glycolysis/gluconeogenesis, and oxidative phosphorylation (Figure 7b). Regardless of carbon chain length, CPs inhibited the conversion of butanal to butanoyl-CoA and of acetoacetyl-CoA to (R)-3-Hydroxy-butanoyl-CoA in the butanoate metabolism pathway. These disruptions may lead to the intracellular accumulation of toxic aldehydes and disrupt cellular energy homeostasis, thereby exacerbating oxidative stress and cellular damage, and ultimately inhibiting algal growth. Furthermore, LCCPs’ exposure suppressed the generation of acetyl-CoA, which subsequently inhibited the synthesis of succinate and fumarate in the TCA cycle. The reduction in acetyl-CoA directly limits its entry into the TCA cycle, thereby impairing the production of ATP and other essential intermediates for biosynthetic pathways [46]. CPs with different carbon chain lengths also suppressed the interconversion of Fructose-1,6P_2_ and Fructose-6P in the glycolytic/gluconeogenic pathways, leading to impaired cellular energy supply and dysregulation of the carbon metabolic network. Notably, LCCPs treatment directly inhibited the oxidative phosphorylation pathway, compromising the ATP synthesis rate and exerting a significant impact on the photosynthesis and growth of *M. aeruginosa*.

## 4. Conclusions

This study systematically elucidates the toxicity and mechanisms of CPs with different carbon chain lengths in the eukaryotic algae *Chlorella* sp. and the prokaryotic algae *M. aeruginosa*. The results demonstrate that the toxicity of CPs is jointly determined by carbon chain length and species-specific traits. CPs with different chain lengths exhibited markedly different toxicities toward microalgae. SCCPs showed the strongest overall toxicity to both algal species, while LCCPs exerted the most pronounced inhibitory effects on Chl-a content in *Chlorella* sp. and *M. aeruginosa*. The eukaryotic *Chlorella* sp. exhibited a stronger comprehensive stress resistance to CPs than the prokaryotic *M. aeruginosa*. Furthermore, CPs’ exposure disrupted cellular structures such as thylakoids, leading to impaired physiological functions and altered antioxidant defense responses. CPs also induced significant species-specific transcriptional responses, triggering DNA damage pathways in *Chlorella* sp. In contrast, CPs perturbed the energy and carbon metabolism networks of *M. aeruginosa* and altered the synthesis and extracellular release of MC-LR and MC-RR. Overall, under CPs exposure, eukaryotic *Chlorella* sp. was primarily affected in DNA replication and photosynthesis, whereas prokaryotic *M. aeruginosa* mainly showed downregulation of metabolic pathways such as glycolysis/gluconeogenesis and oxidative phosphorylation. This study elucidates the molecular response mechanisms of algae with distinct cellular structures to CPs of different carbon chain lengths. These findings provide scientific support for chain length-based ecological risk assessment of CPs and the development of water pollution control strategies. Future studies should extend the exposure duration beyond 96 h to evaluate chronic toxicity and potential physiological adaptations of algae to CP contamination. Long-term experiments will provide insights into whether algae can acclimate to CP stress and whether toxicity intensifies with prolonged exposure.

## Data Availability

The original contributions presented in this study are included in the article/Supplementary Materials. Further inquiries can be directed to the corresponding authors.

## Supplementary Materials

The following supporting information can be downloaded at https://www.mdpi.com/article/10.3390/toxics14040311/s1, Text S1. Analysis of the growth inhibition rate; Text S2. Determination of photosynthetic pigment content; Text S3. SEM and TEM pre-treatment procedures; Text S4. Comparative analysis of comprehensive stress resistance of algae; Text S5. Transcriptomic data processing; Figure S1. Effects of chlorinated paraffins (CPs) of different carbon chain lengths on Microcystin-LR (MC-LR, a) and Microcystin-RR (MC-RR, b) contents in *M. aeruginosa*; Table S1. Rationale for concentration settings; Table S2. Experimental treatment groups; Table S3. Assessment of the comprehensive stress resistance of *Chlorella* sp. and *M. aeruginosa* following exposure to CPs of different carbon chain lengths.
