# Supplementary material for: Toxicity Mechanism of Chlorinated Paraffins with Different Carbon Chain Lengths to Chlorella sp. and Microcystis aeruginosa
Source: Toxics. 2026 Apr 4;14(4):311. doi: 10.3390/toxics14040311 (PMC13119944; doi:10.3390/toxics14040311)
Supplement: Supplementary file 1 [file toxics-14-00311-s001.zip › toxics-4190480-supplementary.pdf]

Contents

Texts

Text S1 Analysis of the growth inhibition rate

Text S2 Determination of photosynthetic pigment content

Text S3 SEM and TEM pre-treatment procedures

Text S4 Comparative analysis of comprehensive stress resistance of algae

Text S5 Transcriptomic data processing

Figures

Figure S1 Effects of chlorinated paraffins (CPs) of different carbon chain lengths on Microcystin-LR (MC-LR, a) and Microcystin-RR (MC-RR, b) contents in *M. aeruginosa*.

Tables

Table S1 Rationale for concentration settings

Table S2 Experimental treatment groups

Table S3 Assessment of the comprehensive stress resistance of *Chlorella* sp. and *M. aeruginosa* following exposure to CPs of different carbon chain lengths

Text S1 Analysis of the growth inhibition rate

The growth inhibition rate was calculated using the following formula [1]:

Growth inhibition rate= $\left(\frac{C_t\text{-}D_t}{C_t}\right)\times 100\%$................................... ....(1)

Where C_t denotes the algal density of the control group at moment *t* (cells/mL), and D_t signifies the algal density of the treatment group at moment *t* (cells/mL).

Text S2 Determination of photosynthetic pigment content

The concentration of chlorophyll-a (Chl-a) was calculated according to the following equations [1].

$$C_{Chl-a} = 12.7 \times OD_{664} - 2.79 \times OD_{647} \quad (2)$$

Text S3 SEM and TEM pre-treatment procedures

Text S3.1 SEM pre-treatment procedure

Algal suspensions were collected and transferred into 2.0 mL centrifuge tubes containing 2.5% glutaraldehyde. The samples were fixed at 4°C overnight. The fixative was then removed, and the samples were washed three times with 0.01 M phosphate-buffered saline (PBS, pH 7.4), 15 min per wash. Dehydration was performed using an ascending ethanol series (30%, 50%, 70%, and 90% ethanol), with 15 min at each concentration, followed by 100% ethanol for 20 min. Finally, the ethanol was replaced with fresh 100% ethanol, and the samples were stored in it. After drying, the samples were sputter-coated with gold for 2 min and observed by scanning electron microscope (SEM, Hitachi Model TM-1000, Japan).

Text S3.2 TEM pre-treatment procedure

Algal suspensions were collected and transferred into 2.0 mL centrifuge tubes containing 2.5% glutaraldehyde. The samples were fixed at 4°C overnight. The primary fixative was removed, and the samples were washed three times with 0.01 M phosphate-buffered saline (PBS, pH 7.4), 15 min per wash. Post-fixation was performed in 1% osmium tetroxide for 1-2 h. The osmium tetroxide solution was carefully discarded, and the samples were washed three times with 0.1 M phosphate buffer (pH 7.4), 15 min per wash. The samples were dehydrated sequentially in 30%,

50%, 70%, and 90% ethanol, with 15 min at each concentration. This was followed by two treatments with 100% ethanol (20 min each) and two changes of 100% acetone (20 min each). After repeated dehydration, the specimens were infiltrated with acetone: EMBed at ratios of 1:1 for 3 h and 1:3 for 4 h, then left overnight in pure EMBed. The embedded samples were ultra-sectioned to 70-90 nm thickness, stained with lead citrate and 50% ethanolic uranyl acetate for 10 min each, and examined using a transmission electron microscope (TEM, Hitachi H-7650, Japan).

Text S4 Comparative analysis of comprehensive stress resistance of algae

The calculation formulas for indicators positively correlated with stress resistance (e.g., SOD activity, CAT activity, GST activity, POD activity, GSH content, Chl-a content) are given as Equation (3), while the formula for the negatively correlated indicator (e.g., MDA content) is given as Equation (4). The membership values of all indicators are then summed to obtain a comprehensive stress resistance index for each species, with a larger comprehensive value indicating stronger stress resistance[2, 3].

$$X(\mu) = \frac{X_0 - X_{min}}{X_{max} - X_{min}} \tag{3}$$

$$X(\mu') = 1 - \frac{X_0 - X_{min}}{X_{max} - X_{min}}$$

(4)

Here, X(μ)和 X(μ') represent the membership values, X_0 denotes a measured value of a given indicator (in this study, the mean value was used), X_{max} is the maximum measured value of the corresponding indicator, and X_{min} is the minimum measured value of the corresponding indicator.

Text S5 Transcriptomic data processing

Text S5.1 Strand-specific transcriptome sequencing (prokaryotic)

Total RNA was extracted from tissue samples. RNA concentration and purity were assessed using a NanoDrop 2000, RNA integrity was evaluated by agarose gel electrophoresis, and the RNA integrity number (RIN) was determined using Agilent 2100. For a single library, the required total RNA amount was 2 μg, with a concentration ≥ 100 ng/μL and $OD_{260/280}$ between 1.8 and 2.2. Ribosomal RNA (rRNA) depletion instead of poly(A) purification is performed by RiboCop rRNA

Depletion Kit for Mixed Bacterial Samples (lexogen, USA), and then all mRNAs were broken into short (300nt) fragments by adding fragmentation buffer first. Secondly, double-stranded cDNA was synthesized with random hexamer primers (Illumina). When the second strand cDNA was synthesized, dUTP was incorporated in place of dTTP. Then the synthesized cDNA was subjected to end-repair, phosphorylation, and 'A' base addition according to Illumina's library construction protocol. RNA-seq transcriptome library was prepared following Illumina® Stranded mRNA Prep, Ligation (San Diego, CA) using total RNA. The paired-end RNA-seq library was sequenced with Illumina NovaSeq X Plus (Illumina Inc., San Diego, CA, USA). The processing of original images to sequences, base-calling, and quality value calculations. The clean reads by removing low-quality sequences, reads with more than 10% of N bases (unknown bases), and reads containing adaptor sequences. The data generated from the Illumina platform were used for bioinformatics analysis.

Text S5.2 De novo eukaryotic transcriptome sequencing

Total RNA was extracted from tissue samples. RNA concentration and purity were evaluated using NanoDrop 2000, RNA integrity was checked by agarose gel electrophoresis, and the RNA quality number (RQN) was measured using Agilent 5300. For a single library, the required total RNA amount was 1 μg, with a concentration ≥ 30 ng/μL, RQN > 6.5, and $OD_{260/280}$ between 1.8 and 2.2. mRNA was enriched from total RNA using oligo(dT)-coated magnetic beads via poly(A)-T base pairing. A fragmentation buffer was added to randomly shear mRNA into ~300 bp fragments. First-strand cDNA was synthesized using random primers and reverse transcriptase, followed by second-strand synthesis to generate stable double-stranded cDNA. The resulting double-stranded cDNA, which initially has sticky ends, was end-repaired to generate blunt ends using an End Repair Mix, and a single adenine (A) base was added to the 3′ ends to facilitate subsequent adapter ligation. Adapter-ligated products were purified and size-selected; the selected fragments were PCR-amplified and purified to obtain the final sequencing library. Libraries were sequenced on an Illumina NovaSeq X Plus platform. Raw sequence data were subjected to quality assessment and filtering. All quality-filtered reads were de novo assembled into

contigs and singletons. Gene expression levels were calculated based on the read counts of clean reads mapped to gene regions.

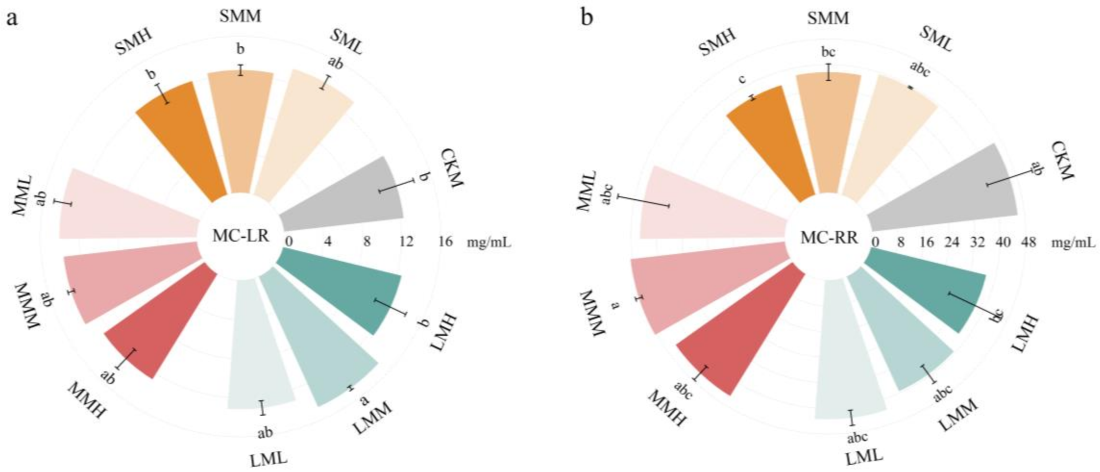

Figure S1 Effects of chlorinated paraffins (CPs) of different carbon chain lengths on Microcystin-LR (MC-LR, a) and Microcystin-RR (MC-RR, b) contents in *M. aeruginosa.*

Table S1 Rationale for concentration settings

	Pollutant	Concentration	Subject	DOI
Actual environmental monitoring	SCCPs	ND-70.3 μg/L; Average: 9.1 μg/L	Groundwater	https://doi.org/10.1016/j.watres.2021.117605
	SCCPs	1.56~56.31 μg/L; Average: 7.22 μg/L	Baiyangdian Lake	https://kns-cnki-net-443.webvpn.las.ac.cn/KCMS/detail/detail.aspx?dbname=CMFD201702&filename=1017149337.n.
	SCCPs	1.131~65.64 μg/L; Average: 18.99 μg/L	Yangtze River	
Toxicology experiment	SCCPs	1, 5, 10, 50, 100, 500, and 1000 μg/L	*Oryzias melastigma*	https://doi.org/10.1016/j.jhazmat.2025.138531
	SCCPs	0, 0.1, 1, 10, 100, and 1000 μg /L	*Brachionus manjavacas*	https://doi.org/10.1016/j.ecoenv.2025.118086
	SCCPs; MCCPs; LCCPs	1, 10, 100, and 1000 μg/L	HepG2 Cell	https://doi.org/10.1016/j.scitotenv.2019.05.388
	SCCPs	25、50、100、200 and 400 μg/L	Wheat	https://doi.org/10.1016/j.jhazmat.2023.132954
	SCCPs	1, 5, 10, 50, 100, and 200 μg/L	Zebrafish embryos/larvae	https://doi.org/10.1016/j.scitotenv.2017.11.304
	SCCPs; MCCPs	0, 1, 5, 10, 50, 100, and 200 μg/L	Zebrafish larvae	https://doi.org/10.1016/j.scitotenv.2024.171372
	SCCPs	99.7, 202, 199, and 199 μg/L	Pumpkin and Soybean Seedlings	https://doi.org/10.1021/acs.est.9b01215

Table S2 Experimental treatment groups

Pollutant	Concentration (mg/L)	Algae	Group
Control	0	*Chlorella* sp.	CKC
Control	0	*Microcystis aeruginosa*	CKM
SCCPs	0.01	*Chlorella* sp.	SCL
	0.1		SCM
	1.0		SCH
	0.01	*Microcystis aeruginosa*	SML
	0.1		SMM
	1.0		SMH
MCCPs	0.01	*Chlorella* sp.	MCL
	0.1		MCM
	1.0		MCH
	0.01	*Microcystis aeruginosa*	MML
	0.1		MMM
	1.0		MMH
LCCPs	0.01	*Chlorella* sp.	LCL
	0.1		LCM
	1.0		LCH
	0.01	*Microcystis aeruginosa*	LML
	0.1		LMM
	1.0		LMH

Table S3 Assessment of the comprehensive stress resistance of *Chlorella* sp. and *M. aeruginosa* following exposure to CPs of different carbon chain lengths

Alage	Treatments	Single index							Comprehensive value	Mean value
		SOD	CAT	GSH	GST	Chl-a	POD	MDA		
Chlorella sp.	SCCPs	0.244	0.317	0.637	0.494	0.503	0.531	0.579	3.305	3.125
	MCCPs	0.562	0.406	0.449	0.494	0.539	0.473	0.573	3.496	
	LCCPs	0.446	0.093	0.287	0.210	0.668	0.368	0.502	2.574	
M. aeruginosa	SCCPs	0.375	0.412	0.416	0.442	0.445	0.381	0.559	3.030	3.088
	MCCPs	0.398	0.458	0.532	0.339	0.352	0.412	0.657	3.148	
	LCCPs	0.326	0.524	0.453	0.507	0.469	0.454	0.352	3.085	

References

[1] LIU X, LI Y, ZHENG X, et al. Anti-oxidant mechanisms of Chlorella pyrenoidosa under acute GenX exposure[J]. Science of the Total Environment, 2021,797: 149005.

[2] ZHANG W, GU P, ZHENG X, et al. Ecological damage of submerged macrophytes by fresh cyanobacteria (FC) and cyanobacterial decomposition solution (CDS)[J]. Journal of Hazardous Materials, 2020,401.

[3] LI Q, YU Y, ZHENG X, et al. Comparative physiological and transcriptomic responses of eukaryotic Chlorella sp. and prokaryotic Microcystis aeruginosa to sulfanilamide stress[J]. Environmental Chemistry and Ecotoxicology, 2025,7: 2179-2187.
